# A Multi-Task Road Feature Extraction Network with Grouped Convolution and Attention Mechanisms

**DOI:** 10.3390/s23198182

**Published:** 2023-09-30

**Authors:** Wenjie Zhu, Hongwei Li, Xianglong Cheng, Yirui Jiang

**Affiliations:** 1 School of Computer and Artificial Intelligence, Zhengzhou University, Zhengzhou 450001, China; zwj1998@gs.zzu.edu.cn (W.Z.);; 2 School of Geo-Science & Technology, Zhengzhou University, Zhengzhou 450052, China

**Keywords:** road feature extraction, multi-task learning network, traffic object detection, lane line segmentation, drivable area segmentation, attention mechanisms

## Abstract

To cope with the challenges of autonomous driving in complex road environments, the need for collaborative multi-tasking has been proposed. This research direction explores new solutions at the application level and has become a hot topic of great interest. In the field of natural language processing and recommendation algorithms, the use of multi-task learning networks has been proven to reduce time, computing power, and storage usage in various task coupling cases. Due to the characteristics of the multi-task learning network, it has also been applied to visual road feature extraction in recent years. This article proposes a multi-task road feature extraction network that combines group convolution with transformer and squeeze excitation attention mechanisms. The network can simultaneously perform drivable area segmentation, lane line segmentation, and traffic object detection tasks. The experimental results of the BDD-100K dataset show that the proposed method performs well for different tasks and has a higher accuracy than similar algorithms. The proposed method provides new ideas and methods for the autonomous road perception of vehicles and the generation of highly accurate maps in visual-based autonomous driving processes.

## 1. Introduction

The rapid development of artificial intelligence technology based on deep learning has made it possible to achieve low-cost, vision-based autonomous driving technology. One of the key issues in autonomous driving technology is how to build an efficient environment perception system. Currently, most assisted autonomous driving technologies are based on high-precision maps. However, the generation of such maps often requires the use of multiple sensors for data collection and extensive post-processing work. If a low-cost vision camera can be used to construct a real-time environment perception system, it can greatly reduce the time and cost required for high-precision map generation, and even achieve updates to the map through vehicle networking, thus having more practical application prospects. In addition, the information provided by the three technologies of traffic object detection, lane line segmentation, and drivable area segmentation plays a crucial role in driving the decision making of vehicles [1]. There is still a lot of research space on how to efficiently complete these three tasks.

**Figure 1:**
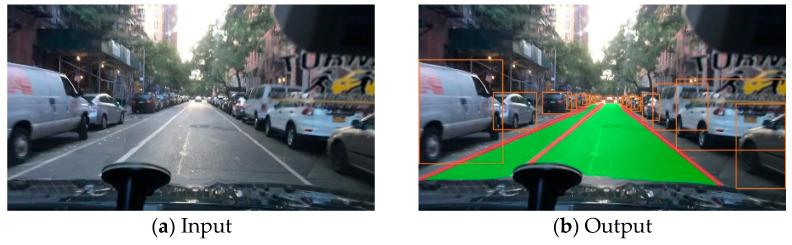
Result of our network.

This paper proposes a multi-task learning network model based on shared encoders and introduces a feature extraction module called C3GC, which can improve the overall accuracy of the model while reducing computational complexity. In addition, we incorporate the transformer and squeeze excitation attention mechanism modules into the model, further improving its accuracy. Extensive experiments on the BDD100K dataset demonstrate the effectiveness of our approach (Figure 1).

The main contributions of this article are as follows:

1. Designing a road feature extraction network model based on multi-task learning, which can simultaneously accomplish three tasks: lane line segmentation, traffic object detection, and drivable area segmentation.
2. Designing an adaptive group convolution module, which can improve the accuracy of the model in this paper without increasing the number of parameters; in addition, the design of a squeeze excitation and transformer attention mechanism module, which effectively improves the accuracy of the model in this paper.
3. Extensive experiments were conducted on the BDD100K dataset, which demonstrated the effectiveness of the proposed method in this paper. Moreover, compared to similar algorithms, the accuracy has been significantly improved.

## 2. Related Work

The three tasks of traffic object detection, lane segmentation, and drivable area segmentation have been extensively studied in their respective fields. In terms of object detection, a series of two-stage object detection algorithms represented by RCNN [2], Fast-RCNN [3], and one-stage object detection algorithms represented by the YOLO series [4,5,6,7] have been developed. The two-stage methods have higher accuracy but poor real-time performance, while the one-stage methods have slightly lower accuracy but are more suitable for real-life production needs because of their high real-time performance. In terms of lane segmentation and drivable area segmentation, traditional segmentation algorithms have been rapidly replaced by large-scale applications of convolutional neural networks in recent years. Many high-performance segmentation models have been developed, such as the encoder–decoder structure in UNET [8] and the feature pyramid structure used in FPN [9], which allows the network to obtain features of different scales, greatly improving the accuracy of the segmentation network. In addition, the RESA [10] method, SCNN [11] method, and Lanenet [12] method have also shown excellent results in the field of lane segmentation.

In the application scenarios of autonomous driving, multiple tasks usually need to work simultaneously to provide services. Considering that the computing resources of the onboard computer are limited and there is a high real-time requirement, it is impractical to set up separate models for each task. Therefore, a method is needed to couple these similar tasks together to enable them to use fewer resources while maintaining real-time requirements. Multi-task learning networks [13] provide an effective solution to this problem, allowing related tasks to share the use of feature extraction networks, thereby saving time, space, and resources. Its effectiveness has been proven in the field of natural language processing and recommendation algorithms [14,15,16]. In the field of computer vision, many models adopt the idea of Faster RCNN [17] and use the ResNet [18] structure for feature extraction. This fully demonstrates its powerful feature extraction ability, which can meet the needs of multi-task learning networks. LSNet [19], MultiNet [20], YOLOP [21], and HyBridNet [22] all use a ResNet-based shared encoder, and the results show that multi-task learning networks can simultaneously complete multiple related tasks with only a small increase in time and resource consumption.

## 3. Methodology

This paper adopts a multi-task learning method based on shared encoders for the network. In the Backbone part, a unified network model based on the improved YOLOv5s Backbone was used. Multiple attention mechanisms were integrated in the Neck part, and group convolution was also adopted to improve computational efficiency. Afterwards, unique decoder heads were set for different branch tasks, making it possible to simultaneously execute multiple tasks. In this section, we will introduce the structure and related parameters of the multi-task learning road feature extraction network used in this article. In the first subsection, we will introduce the implementation of its shared feature extraction module and discuss how it works collaboratively to complete traffic object detection, lane segmentation, and drivable area segmentation tasks. The second subsection will describe the calculation method of the loss function in our method and how to use it to control the weight of different tasks.

### 3.1. Network Structure

**Figure 2:**
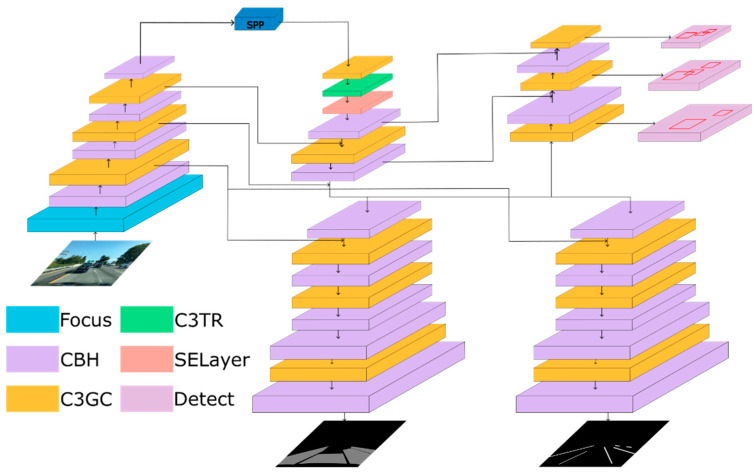
The architecture of our network.

Previous research, such as YOLOP and HybridNet, has shown that the current mainstream feature extraction networks, such as Darknet [7], can perform well in feature extraction tasks. Therefore, in this article, the main structure of the backbone network adopts a similar design concept. However, transformer [23] and SE attention mechanism [24], as well as a grouped convolution structure, were added on this basis. These additions were used to make the feature extraction in the backbone network more efficient. In the decoder head, the FPN idea was also used in this article, connecting the features of some layers in the backbone network to the output part of the decoder. The detailed network structure is shown in Figure 2. 

#### 3.1.1. Backbone

**Figure 3:**
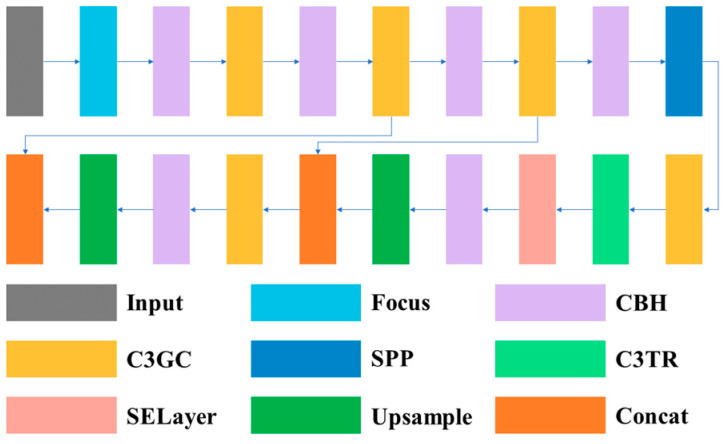
The architecture of the backbone.

The backbone network of this article’s network is mainly composed of CBH and adaptive group convolution modules. The original RGB image was inputted and alternately passed through the CBH and adaptive group convolution modules, and the extracted feature map was further extracted through the SPP [25] module in the neck part and the transformer attention module, SELayer module, to perform feature extraction. Afterwards, the extracted features were upsampled multiple times to obtain the final feature map, which was provided to the decoder heads of different tasks. The structure of the backbone is shown in Figure 3.

**Figure 4:**
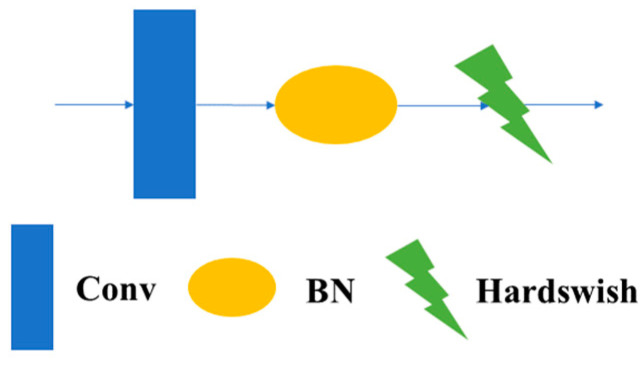
Structure of CBH.

3.1.1.1 CBH Module: The CBH module in this article consists of convolutional blocks, a BatchNorm2d module, and a Hardswish activation function. The formula of the Hardswish activation function is shown in Formula (1), and the structure of the CBH module is shown in Figure 4.

(1) $$Hardswish\left(x\right)=\left\{\begin{array}{c} x\;\;\;\;\;\;\;\;\;\;\;\;\;\;\;x>3\\ 0\;\;\;\;\;\;\;\;\;\;\;\;x<-3\\ \frac{x^{2}+3x}{6}\;\;\;\;\;otherwise \end{array}\right.$$

**Figure 5:**
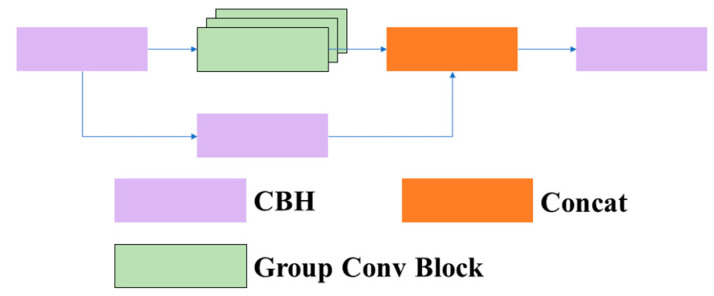
Structure of the group convolution block.

**Figure 6:**
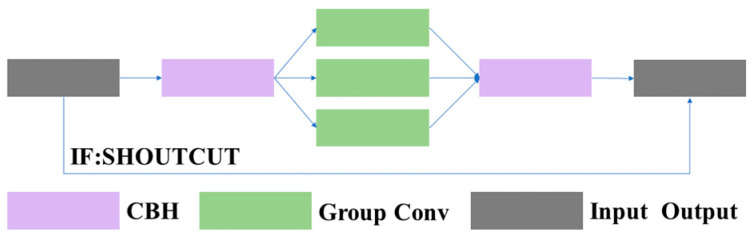
Structure of the adaptive group convolution block.

3.1.1.2 C3GC: In this article, we replaced the common BottleneckCSP [26] module with the C3 module and added an adaptive grouped convolution module [27] based on the C3 module, which we named C3GC. The structure of the C3GC module is shown in Figure 5. This module mainly modifies the Bottleneck structure in the C3 module to our designed adaptive convolution module. This module consists mainly of two CBH layers and an adaptive group convolutional layer. The structure of the adaptive convolution module is shown in Figure 6.

**Figure 7:**
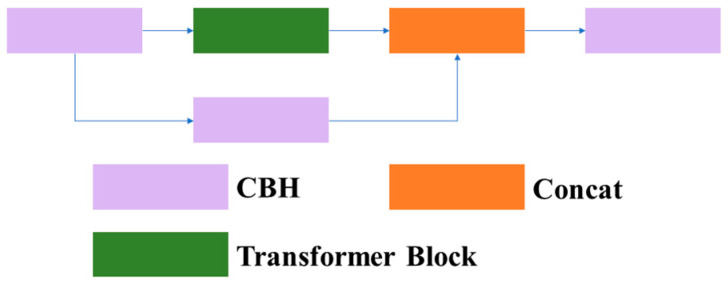
Structure of C3TR.

**Figure 8:**
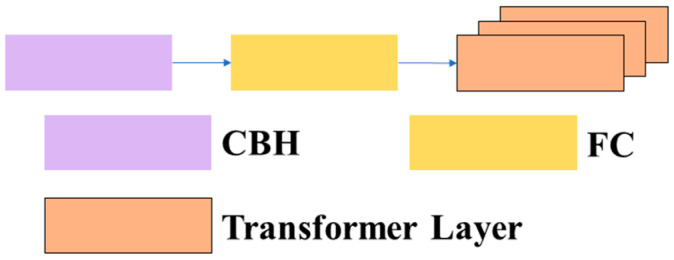
Structure of the transformer block.

**Figure 9:**
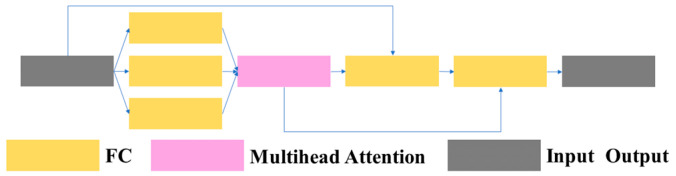
Structure of the transformer layer.

3.1.1.3 C3TR: Due to the interpretability, efficiency, and scalability of the transformer, and its frequent application in contextually related scenarios between different positions, tthis article introduces the C3TR module, which combines the transformer module with the C3 module by replacing the bottleneck structure in the traditional C3 module with a transformer block structure. This article refers to this structure as C3TR. The structures of the C3TR module, transformer block module, and transformer layer are shown in Figure 7, Figure 8 and Figure 9.

**Figure 10:**
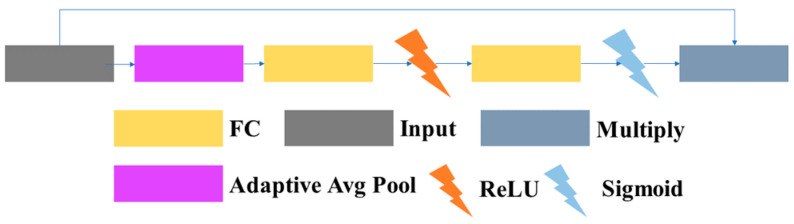
Structure of the SELayer.

3.1.1.4 SELayer: The SELayer, also known as the channel attention module, is referred to as the SELayer in this article. Due to the often consistent color of the lane and lane markings, the SE attention can adaptively learn the importance of each color channel to improve the performance of the model. Therefore, this paper introduces the SE attention mechanism to enhance the ability to extract lanes and lane markings. The structure used in this article is similar to the structure in SENet. It consists of a global average pooling layer, two fully connected layers, and parts that use ReLU and sigmoid activation functions. The structure of the SELayer module is shown in Figure 10.

#### 3.1.2. Decoder Head

In terms of traffic object detection tasks, considering that the YOLOv5 object detection network already has high performance, this article retains the design scheme of YOLOv5 and adopts an anchor-based multi-scale detection scheme. The bottom-up feature aggregation network is combined with the feature pyramid network, and then assigned to anchor points of different scales for object detection.

We found through experimentation that, in terms of lane and crosswalk segmentation, using only features extracted from the last layer of the neck in the YOLOP method resulted in lower accuracy. Therefore, in this article, we decided to design a decoder head for these two tasks based on their characteristics.

**Figure 11:**
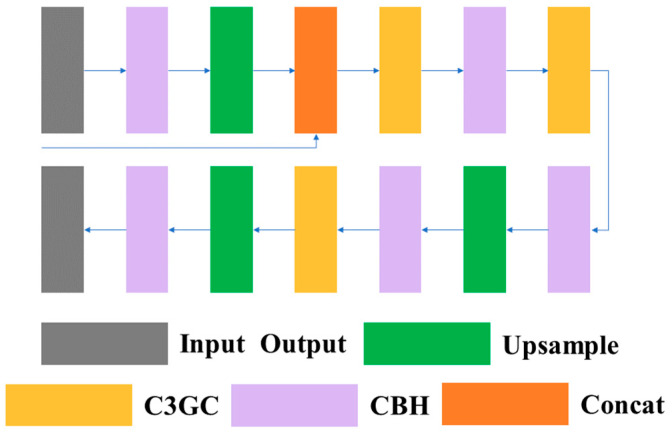
The architecture of the decoder head.

This paper takes into account the long and scattered characteristics of lane line in the design of lane line segmentation tasks, which often span multiple convolution blocks and cannot extract enough features in larger convolution blocks, resulting in the loss of semantic information in the feature extraction process. Therefore, this paper adopted the idea of FPN (Feature Pyramid Network). It combines the shallow low-level semantic information of the first two layers in the main network with the results obtained in Section 3.1.1 before upsampling. This allows the decoder head to better identify some small-scale semantic information that disappears during convolution. The design of the decoder head is shown in Figure 11.

In terms of segmentation in the drivable area, this article found that using a decoder head similar to the lane line segmentation task can also improve accuracy. As the area of the drivable region is large but the edge area is irregular, most algorithms have poor extraction performance in the edge area. Similar designs can effectively improve segmentation performance in the edge area. However, since the accuracy of similar networks in this task is already high, the improvement in this article is limited, and this will result in a loss of about 0.003 s of inference time per frame.

### 3.2. Loss Function

For multitask learning networks, a common method for setting the loss function is to independently calculate the losses for different tasks and then perform a weighted average. The calculation method for the overall loss *L_all_* is shown in Formula (2).

(2) $$L_{all}=\alpha_{1}L_{det}+\alpha_{2}L_{da}+{\alpha_{3}L}_{ll}$$

Formula (1) contains the loss function *L_det_* which is composed of three parts, classification loss *L_class_*, object loss *L_obj_* and bound-ing box loss *L_box_*, specifically designed for the traffic object detection task. The computation of the loss function is performed through weighted averaging, as shown in Formula (3).

(3) $$L_{det}=\beta_{1}L_{class}+\beta_{2}L_{obj}+{\beta_{3}L}_{box}$$

In Formula (2), *L_class_* and *L_obj_* are focal losses used to evaluate the quality of classification, and *L_box_* is used to measure the similarity between the generated predicted boxes and the actual values. The calculation method of *L_box_* adopts the calculation method of LIoU.

The loss for *L_da_* in the task of drivable area segmentation and *L_ll_* in the task of lane line segmentation in Formula (1) are calculated using the traditional segmentation loss calculation method, the cross-entropy loss function LCE. 

Finally, the total loss is obtained by summing up these losses with weights *α*_1_, *α*_2_, *α*_3_, *β*_1_, *β*_2_, *β*_3_, corresponding to each part of the loss in Equations (1) and (2). In multi-task learning networks, the weight setting between different tasks is often controlled by the weight of the loss function, so the weight of different tasks has a great impact on the accuracy of different tasks in the network. However, similar algorithms such as YOLOP and HybridNet do not consider the impact of weight setting on the final network accuracy. However, in our previous research work, suitable task weight allocations for this type of multi-task learning task have already been obtained. Specifically, for the traffic object detection task, it was less sensitive to the weight size, followed by lane segmentation tasks, while lane line segmentation tasks were the most sensitive to weight changes. Therefore, the emphasis of the weight setting in this paper is on the lane line and road segmentation tasks, while the weight setting for the traffic object detection task is relatively low.

## 4. Experimental Section

This article primarily focuses on three aspects in the experimental section. Firstly, it introduces the relevant settings of the experiments and the use of the dataset. Secondly, it compares this paper’s method with similar methods from three different branches of tasks. Lastly, it conducts ablation experiments, primarily exploring the effectiveness of different modules in this paper’s method and comparing multitasking with single-tasking to validate the effectiveness of the multitasking approach.

### 4.1. Experimental Setup

#### 4.1.1. Dataset

**Figure 12:**
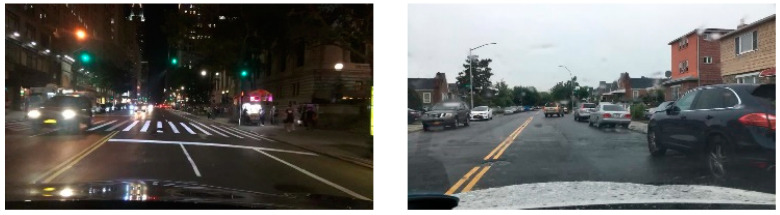
Example of a BDD100K dataset.

In terms of datasets, this article used the BDD100K dataset [28]. The BDD100K dataset is one of the more comprehensive datasets for the autonomous driving field produced in recent years, containing 100 K frames of images and 10 task annotations related to autonomous driving direction, making it suitable for researching multi-task learning networks. In addition, due to the large amount of data in the dataset, it has geographical, temporal, and weather diversity, which makes the network trained in this article on the dataset highly generalizable. Moreover, this dataset is often used in similar methods, so using it in this article facilitates performance comparisons with similar methods. In terms of dataset partitioning, this article extracted 70 K images from the 100 K image dataset as the training set, 20 K as the validation set, and the remaining 10 K data as the test set. An example of a dataset is shown in Figure 12.

#### 4.1.2. Parameters and Experimental Setup

In terms of parameter settings, this article uses the Adam optimizer for model training and uses the warm-up and annealing algorithm to adjust the learning rate, ensuring that the model can converge better. This ensures that this article can study the impact of multi-task weight settings on the final accuracy of multi-task learning networks under the same conditions. The specific parameter settings are shown in Table 1.

In terms of experiments, this paper not only compares the performance with existing multitask learning methods, but also selects some excellent methods focusing on single tasks, all of which have achieved excellent performance on the BDD100K dataset. Examples of these methods include YOLOv5s and Faster-RCNN, which are representatives of one-stage and two-stage object detection algorithms, respectively. PSPNet [29] is a representative method in the field of semantic segmentation. Since there have not been many lane segmentation methods applied to the BDD100K dataset, this paper uses excellent methods from other datasets for performance comparison. In addition, in this section, the experiments in our method were conducted under the best weight settings. The specific hardware information and settings are shown in Table 1.

### 4.2. Experimental Results

#### 4.2.1. Traffic Object Detection Results

**Figure 13:**
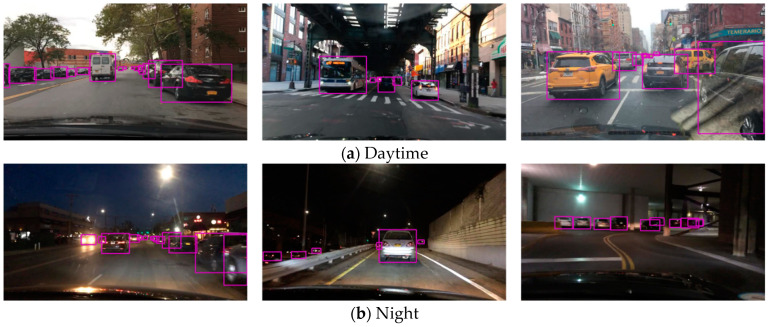
Traffic object detection result.

The visualization of the results of traffic object detection is shown in Figure 13. Considering that similar algorithms can only detect vehicle objects, this paper also only considers the detection results of vehicle objects on the BDD100K dataset in this section. The results are shown in Table 2, with Recall and mAP50 selected as evaluation metrics. The performance results show that the proposed method achieved an accuracy comparable with mainstream object detection methods, although it still had a large gap in real-time performance compared to faster methods such as YOLOv5s. However, the proposed approach was capable of simultaneously completing additional tasks such as drivable area segmentation and lane segmentation. Moreover, it achieved a runtime of only 12.8 ms, reaching 78 fps, which exceeds the commonly used 60 fps output of automotive cameras, thereby meeting the real-time requirement.

#### 4.2.2. Drivable Area Segmentation Result

**Figure 14:**
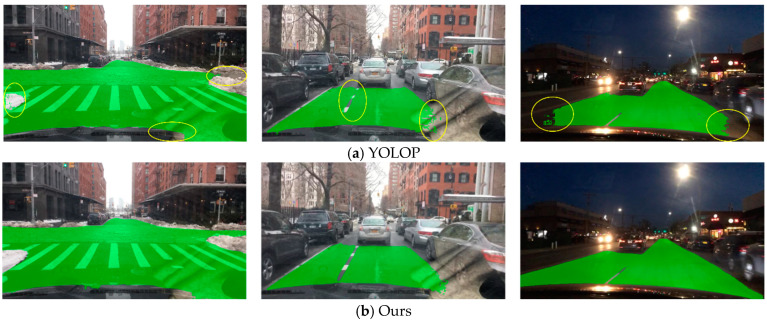
Drivable area segmentation results.

The visualization of the segmented driving area is shown in Figure 14. In this task, our method only needed to segment the area where vehicles can drive from the background (i.e., the road). We used mIoU as the evaluation metric for this task, and the specific evaluation metric data are shown in Table 3. From the results, it can be seen that our method had higher accuracy than similar methods and reached the level of the PSPNet method. It was also faster than similar methods, meeting the real-time requirements. It can be inferred from the results that the structure of our method produced smoother results at the edges and reduced the results produced in the opposite lane, resulting in higher accuracy on the test set.

#### 4.2.3. Lane Line Segmentation Result

**Figure 15:**
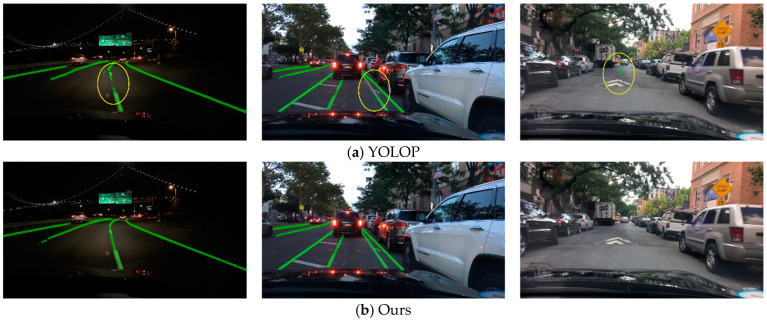
Lane line segmentation result.

The visualization of the lane line segmentation results is shown in Figure 15. In this task, the accuracy and lane IoU were used as evaluation metrics. The specific results are shown in Table 4, which indicate a significant improvement in the performance of our method compared to the compared methods, reducing the phenomenon of lane line interruption during the detection process. Compared to the YOLOP method used as the baseline, our improved method achieved an 8.2% increase in accuracy in the lane line segmentation task. Although the added structure in our method increased the additional inference time compared to the baseline, it still met the real-time requirements.

#### 4.2.4. Experiment Conclusion

Through the experiments above, it can be observed that the method proposed in this paper achieved an accuracy similar to task-specific methods in single-task scenarios. Additionally, compared to the YOLOP method in the context of multi-task approaches, our method showed significant improvement in lane line segmentation and road segmentation. In terms of lane line segmentation, there was a significant reduction in the number of lane line interruptions compared to YOLOP. There was also a substantial improvement in road edge segmentation compared to YOLOP. Although there was still an accuracy gap when compared to the HybridNets method, it could only achieve approximately 35 fps, indicating poor real-time performance.

### 4.3. Ablation Experiment

This section verifies the effectiveness of the proposed method through three sections of ablation experiments. In the first part, we will verify the effectiveness of adaptive convolutional structures. In the second part, we will compare experiments to validate the effectiveness of the C3TR and SE structure. In the third part, we will perform experiments comparing multi-task and single-task networks to verify the effectiveness of multi-task networks.

#### 4.3.1. Adaptive Convolutional Block

In this section, we only conducted experiments using the C3GC module, and the results of the experiments are shown in Table 5. From the results, it can be observed that adding the C3GC module on top of the Baseline+C3TR module improved the accuracy of the lane segmentation task by about 0.4%. Although there was no significant improvement in accuracy for other tasks, we found that during training, using this module accelerated the model convergence without increasing the inference time. Therefore, the C3GC module in this paper can be considered effective.

#### 4.3.2. Attention Mechanism

In this section, we conducted experiments based on Section 4.3.1, adding the transformer attention module and the SE attention module. From the results, it can be observed that the transformer attention mechanism module C3TR significantly increased the accuracy of the model, achieving a 0.1% and 0.2% accuracy improvement in object detection and drivable area segmentation tasks, respectively. The improvement was more noticeable in the lane segmentation task, with a 4.5% increase in accuracy and a 0.2% increase in Lane IoU. Although the SE attention module may not show significant improvements or even a decrease in accuracy of about 0.1% in tasks like object detection and drivable area segmentation, its enhancement in lane line segmentation is evident. Compared to previous methods, the module improved Acc by 0.9% and Lane IoU by 0.6%. These experiments demonstrate the effectiveness of the two attention mechanism modules described in this paper. In addition, calculations were also made on the parameter amount and running time of different structural models in the experiment. From the time results, it can be observed that although adding additional structures would result in extra computations and increase the running time, it can still meet the real-time requirement. In terms of parameter amount, the C3TR module increased the parameter amount by approximately 1.18 M and the SELayer increased it by approximately 0.13 M, while using the C3GC module could reduce the parameter amount by 1.91 M. Therefore, after adding additional modules, our method had a reduced parameter amount compared to the baseline. This indicates that our method is feasible in terms of computational complexity and usability.

#### 4.3.3. Multitask vs. Single Task

This section of the article verifies the effectiveness of the multi-task approach by comparing it with the single-task approach. Performance data for executing both the single and multi-task approach using the network are shown in Table 6. From the performance data, it can be observed that implementing the multi-task model can achieve even higher accuracy than executing a single task, while also saving a lot of time. This phenomenon is due to the faster decline of the loss function for the target detection task during the training process, which allows for quick convergence. Additionally, the use of a shared encoder further allows the network to remain in a pre-training state after the target detection task has converged, which can then be utilized to improve accuracy for the other two tasks that converge at a slower rate through the remainder of the training. The evaluation metrics and relevant settings for the ablation experiments are consistent with those outlined in the aforementioned content.

## 5. Conclusions

This article proposed a multi-task road feature extraction network that combines transformer and SE attention mechanism and adaptive group convolution blocks, and applies a FPN-like structure in its decoder head. These efforts enable the multi-task learning network to efficiently perform complete traffic object detection, lane segmentation, and drivable area segmentation tasks. At the end, the proposed method achieved 74.9% accuracy and 27.7% Lane IoU in the lane segmentation task, 75.8% mAP50 in the traffic object detection task, and 97.6% accuracy and 91.9% mIoU in the drivable area segmentation task. Compared to similar algorithms, the method proposed in this paper achieved an 8% improvement in accuracy for lane line segmentation tasks and a 1% improvement in accuracy for drivable area segmentation tasks, while maintaining a comparable level of accuracy for traffic object detection tasks. Considering the characteristics of the backbone network in this study, more branch tasks can be added in the future to further enhance the method’s flexibility. This method combines multitask learning networks with high-precision semantic segmentation and detection tasks, and can reduce the reliance on expensive sensors in autonomous driving and assisted driving. The low-cost and high-precision visual road feature extraction algorithms are widely applied, and they can even be combined with technologies such as cloud computing and digital twins to share and quickly update information. In addition, this approach can be further expanded to cover a wider range of tasks, thus involving different usage scenarios and providing more ideas and solutions for future real-time tasks, such as autonomous driving.

## Figures and Tables

**Table 1:** Experimental Setup.

Parameters	Strategy
GPU	Nvidia RTX 2070 Super
α1, α2, α3	1.1, 0.5, 0.8
β1, β2, β3	0.35, 0.7, 0.05
LR0	0.0002
CLR	0.5
Epoch	110
Batchsize	140
C3GC1-4	Inchannel: 64–512 Outchannel: 64–512
Group Size	4
C3TR	Inchannel: 512 Outchaanel: 512
SELayer	Channel: 512 R: 4

**Table 2:** Traffic object detection results.

Network	Recall	mAP50	Speed (ms)
MultiNet [20]	81.3	60.2	30.5
Faster R-CNN	81.2	64.9	29.8
YOLOv5s	86.8	77.2	3.2
YOLOP	89.2	76.5	6.4
HybridNets	92.8	77.3	28.2
Ours	89.1	75.8	12.8

**Table 3:** Drivable area segmentation results.

Network	mIoU	Speed (ms)
MultiNet	71.6	30.5
DLT-Net [30]	71.3	-
PSPNet	89.6	23.7
YOLOP	90.9	6.4
HybridNets	90.5	28.2
Ours	91.9	12.8

**Table 4:** Lane line segmentation results.

Network	Accuracy	Lane IoU	Speed (ms)
SCNN [31]	35.79	15.84	13.8
Enet [32]	34.12	14.64	-
R-101-SAD [33]	35.56	15.96	-
ENet-SAD	36.56	16.02	5.2
YOLOP	66.6	26.0	6.4
HybridNets	85.4	31.6	28.2
Ours	74.9	27.7	12.8

**Table 5:** Ablation experiment.

Scheme	Recall	mAP50	DA mIoU	LL Accuracy	Lane IoU	Parameter	Time (ms)
Baseline	89.2	75.6	91.8	69.5	26.5	8.897 M	9.6
C3TR	89.3	75.7	92.0	73.6	26.7	10.08 M	10.1
C3GC+C3TR	89.2	75.7	92.0	74.0	27.1	8.17 M	10.4
Ours	89.1	75.8	91.9	74.9	27.7	8.301 M	12.8

**Table 6:** Multitask vs. single task.

Method	Recall	mAP50	DA mIoU	LL Accuracy	Lane IoU
Det (Only)	89.3	75.6	_	_	_
DA-Seg (Only)	_	_	61.6	_	_
LL-Seg (Only)	_	_	_	56.4	24.2
Multitask	89.1-	75.8+	91.9+	74.9+	27.7+

## Data Availability

Publicly available datasets were analyzed in this study. BDD100K dataset can be found at https://bdd-data.berkeley.edu/.
